# Multiple Sclerosis and the Risk of Cardiovascular Diseases: A Mendelian Randomization Study

**DOI:** 10.3389/fimmu.2022.861885

**Published:** 2022-03-15

**Authors:** Fangkun Yang, Teng Hu, Kewan He, Jiajun Ying, Hanbin Cui

**Affiliations:** ^1^ Department of Cardiology, Ningbo Hospital of Zhejiang University (Ningbo First Hospital), School of Medicine, Zhejiang University, Ningbo, China; ^2^ Department of Cardiology, Second Affiliated Hospital of Zhejiang University, School of Medicine, Zhejiang University, Hangzhou, China; ^3^ Cardiology Center, Ningbo First Hospital, Ningbo University, Ningbo, China; ^4^ School of Medicine, Ningbo University, Ningbo First Hospital, Ningbo, China

**Keywords:** multiple sclerosis, cardiovascular disease, Mendelian randomization, genome-wide association studies, causal association

## Abstract

**Background:**

Observational studies suggested that multiple sclerosis (MS) is associated with cardiovascular diseases (CVDs). However, the causal association has not been fully elucidated. Thus, we aim to assess the causality of the associations of MS with risk of CVDs.

**Methods:**

A two-sample Mendelian randomization (MR) study was performed to explore the causality. Genetic instruments were identified for MS from a genome-wide association study (GWAS) involving 115,803 individuals. Summary-level data for CVDs were obtained from different GWAS meta-analysis studies. MR analysis was conducted mainly using the inverse-variance weighted (IVW) method. Sensitivity analyses were further performed to ensure the robustness of the results.

**Results:**

This MR study found suggestive evidence that genetic liability to MS was associated with an increased risk of coronary artery disease (CAD) [odds ratio (OR), 1.02; 95% confidence interval (CI), 1.00–1.04; *p* = 0.03], myocardial infarction (MI) (OR, 1.03; 95% CI, 1.00–1.06; *p* = 0.01), heart failure (HF) (OR, 1.02; 95% CI, 1.00–1.04; *p* = 0.02), all-cause stroke (AS) (OR, 1.02; 95% CI, 1.00–1.05; *p* = 0.02), and any ischemic stroke (AIS) (OR, 1.02; 95% CI, 1.00–1.05; *p* = 0.04). The null-association was observed between MS and the other CVDs. Further analyses found little evidence of pleiotropy.

**Conclusions:**

We provided suggestive genetic evidence for the causal associations of MS with increased risk of CAD, MI, HF, AS, and AIS, which highlighted the significance of active monitoring and prevention of cardiovascular risk to combat cardiovascular comorbidities in MS patients.

## Introduction

Multiple sclerosis (MS) is one of the most common chronic neurological disorders targeting the central nervous system (1). Characterized most generally by impaired ambulation and slowed cognitive processing, MS is the principal non-traumatic cause of disability (2). Cardiovascular diseases (CVDs), comprising coronary artery disease (CAD), myocardial infarction (MI), heart failure (HF), atrial fibrillation (AF), and stroke, are the leading causes of mortality and morbidity worldwide (3). CVDs account for 30% of all global deaths and place a substantial economic burden on the society (4).

The associations between MS and CVDs drawn from epidemiological studies have attracted much attention in the recent years (5, 6). A population-based retrospective cohort study based on 12,251 patients with MS and 72,572 controls suggested that MS was associated with an increased risk of acute coronary syndrome [hazard ratio (HR), 1.28; 95% CI, 1.09–1.51] (7). MS was also associated with increased stroke risks (HR, 4.93; 95% CI, 2.85–8.55) in a cohort study followed 5 years (8). Similarly, another retrospective study involving 14,565 patients with MS and 72,825 matched controls found that MS contributed to a higher risk of MI (adjusted HR 1.63; 95% CI 1.43–1.87) (9). However, a Swedish cohort study of 7,767 cases and 76,045 controls indicated that MS was associated with a decreased risk of angina pectoris and AF (10). The higher incidence of cardiovascular comorbidity compared with age-matched healthy controls may influence low-, mid- and long-term clinical outcomes, namely, greater general disability status, worse physical outcomes, higher depression scores, cognitive aging, and low satisfaction with quality of life (11). Besides, cardiovascular comorbidity burden was reported to be associated with MRI-derived disease outcomes (such as white matter hyperintensities, gray matter volumes and hippocampal volumes) in MS patients (12, 13). Meanwhile, higher Framingham risk score was associated with higher risk of relapses, disability, and disease-modifying therapy escalation in patients with MS over a 5-year follow-up (14). Traditional observational studies are susceptible to potential confounders and reverse causality bias, making it difficult to infer the causality (15). Therefore, the potential causal relationship between MS and CVDs remains unclear.

The Mendelian randomization (MR) method uses genetic variants as the proxy of the exposure in order to evaluate the causal effect of that exposure on the outcome (16). Since the genetic variants are randomly passed to the offspring and kept constant after conception, they can circumvent confounding factors and diminish interference of reverse causality between exposures and outcomes (17).

Hence, to examine the potential causality of genetic liability to MS with the risk of CVDs, we performed a two-sample MR analysis, leveraging the most updated genome-wide association study (GWAS) data.

## Methods

### Study Design

**Figure 1:**
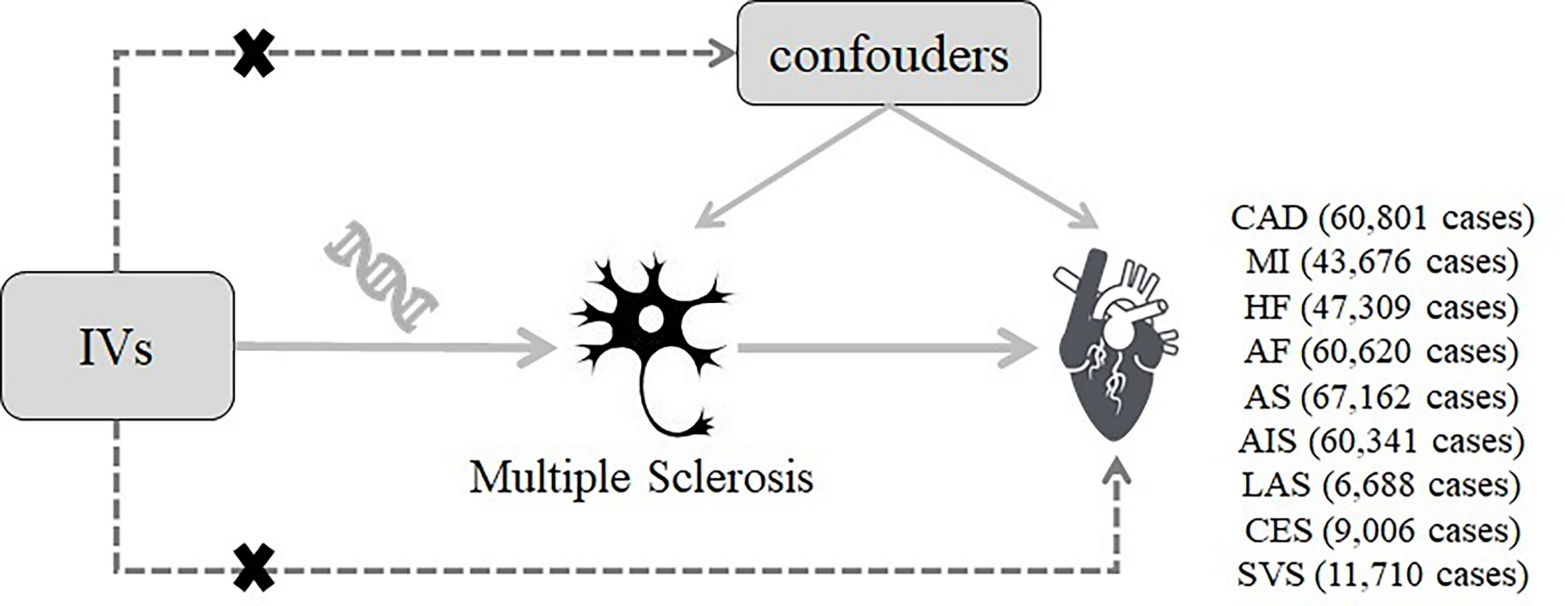
Diagram of the two-sample Mendelian randomization study for the association between multiple sclerosis and the risk of cardiovascular diseases. IVs, instrumental variables; CAD, coronary artery disease; MI, myocardial infarction; HF, heart failure; AF, atrial fibrillation; AS, all-cause stroke; AIS, any ischemic stroke; LAS, large artery stroke; CES, cardioembolic stroke; SVS, small vessel stroke.

The current study was a two-sample MR study based on the genetic data obtained from the worldwide genetic consortia. The conceptual schematic of the current MR study is shown in **Figure 1**. Briefly, genetic variants were used as instrumental variables to investigate the causal associations between MS and CVDs based on three core assumptions. First, the selected genetic variants should be significantly associated with MS (*p <*5 × 10^−8^). Second, the genetic variants should not be associated with any potential known confounders. Third, the genetic variants should influence the CVDs only *via* MS. All original studies included have obtained ethical review approval and informed consent from the participants.

### Instrument Selection

Genetic instrumental variables for MS were extracted from the International Multiple Sclerosis Genetics Consortium, namely, 47,429 MS cases and 68,374 control, which explain ~48% of the estimated heritability for MS (2). The genetic associations with MS were adjusted for confounding factors, namely, use of immunomodulatory drugs, age, gender, batch effects, and the first 10 principal components (accounting for unmeasured confounders). In total, 233 single nucleotide polymorphisms (SNPs) were identified, in which 78 reached genome-wide significance (*p <*5 × 10^−8^). The F-statistics was calculated to assess the strength of each instrument. A threshold of F-statistics >10 indicated that the genetic variants had relatively strong estimated effects in the MR analyses (18). In addition, to ensure that the SNPs were valid and independent (*r* ^2^ <0.01), the linkage disequilibrium (LD) in selected SNPs was calculated based on the 1,000 genome LD reference panel (EUR population). In the current MR study, 68 independent SNPs were identified as instrumental variables for MS after exclusion of ten SNPs in moderate LD (*r* ^2^ >0.01).

### Data Sources for Outcomes

Summary-level data for the associations of MS-associated SNPs with CAD and MI were derived from the Coronary Artery Disease Genome-Wide Replication and Meta-analysis plus The Coronary Artery Disease (CARDIoGRAMplusC4D) 1,000 genome-based GWAS meta-analysis, which involved 60,801 CAD cases (123,504 controls) and 43,676 MI cases (128,199 controls) from 48 cohorts (approximately 77% of participants with European ancestry) (19). Summary-level data for AF were obtained from meta-analyzed GWAS, namely, 1,030,836 participants (60,620 cases and 970,216 controls) from the Nord-Trøndelag Health Study (HUNT), the Collaborative Analysis of Diagnostic Criteria in Europe study (deCODE), the Michigan Genomics Initiative (MGI), the DiscovEHR, the UK Biobank, and the Atrial Fibrillation Consortium (AFGen) Consortium (20). Summary statistics for HF were acquired from the Heart Failure Molecular Epidemiology for Therapeutic Targets (HERMES) Consortium, which contained 47,309 cases and 930,014 control of European ancestry (21). For stroke and ischemic stroke subtypes, summary data were extracted from a multi-ancestry GWAS of 29 studies in the MEGASTROKE consortium involving 67,162 cases of all-cause stroke (AS), 60,341 cases of any ischemic stroke (AIS), 6,688 cases of large artery stroke (LAS), 9,006 cases of cardioembolic stroke (CES), and 11,710 cases of small vessel stroke (SVS) (22). The information of all the genetic datasets used in the current study is displayed in **Table 1**.

**Table 1:** Detailed information of the studies and datasets used for Mendelian randomization analyses.

Phenotype	Consortium	Ethnicity	Sample size	Cases	Year
Multiple Sclerosis	IMSGES	European	115,803	47,429	2019
Coronary Artery Disease	CARDIoGRAMplusC4D	77% European	184,305	60,801	2015
Myocardial Infarction			171,875	43,676	
Atrial Fibrillation	AFGen	European	1,030,836	60,620	2018
Heart Failure	HERMES	European	977,323	47,309	2020
All-cause Stroke	MEGASTROKE	Mixed	514,791	67,162	2018
Any Ischemic Stroke				60,341	
Large Artery Stroke				6,688	
Cardioembolic Stroke				9,006	
Small Vessel Stroke				11,710	

IMSGES, International Multiple Sclerosis Genetics Consortium; CARDIoGRAM-plusC4D, Coronary Artery Disease Genome-Wide Replication and Meta-analysis plus the Coronary Artery Disease; AFGen, Atrial Fibrillation Genetics; HERMES, Heart Failure Molecular Epidemiology for Therapeutic Targets.

### Statistical Analysis

We performed a two-sample MR analysis using the fixed-effects inverse-variance weighted (IVW) method as the main analysis to evaluate the causal associations between MS and CVDs. The genome-wide significant (*p <*5 × 10^−8^) and independent (*r* ^2^ <0.01) SNPs were identified as the genetic instrumental variables of MS from the exposure dataset. Then the genetic associations of MS-related SNPs with CVDs were obtained from corresponding outcome datasets. The Wald ratio method was applied to derive MR estimates of the effect of MS on CVDs, which was the ratio of SNP-outcome genetic effect over SNP-exposure genetic effect (23). This method provided the most robust causal estimates, while was relatively sensitive to pleiotropy (24). Thus, the Weighted median method, the MR-Egger regression, and the MR Pleiotropy Residual Sum and Outlier (MR-PRESSO) method were further applied as supplementary analyses. Specifically, the Weighted median method can generate reliable causal estimates when at least 50% of the weight in the analysis comes from effective SNPs (25). The MR-Egger regression can detect potential pleiotropy and provide estimates after correction for pleiotropy (26). The MR-PRESSO method could detect possible outliers and calculate causal estimates after removing the identified outliers (27). The Cochrane’s Q-derived *p*-value was calculated to evaluate the degree of heterogeneity, where *p <*0.05 indicated horizontal pleiotropy (28). The *p*-value for the intercept in MR-Egger was used to detect the directional pleiotropic effect (26). Furthermore, scatter plots depicting the associations of genetic liability to MS with CVDs were provided. The Bonferroni correction was used to account for multiple testing and association with a *p*-value <0.006 (0.05/9 outcomes) was considered statistically significant. The *p*-values between 0.006 and 0.05 were deemed suggested associations. The specialized web application named mRnd (https://shiny.cnsgenomics.com/mRnd/) was used for the statistical power calculation of MR analyses (29). The main parameters contained the sample size, proportion of cases, odds ratio (OR) of outcome, and proportion of variance explained for the exposure variable (*r* ^2^). All the data analyses were conducted using R Software (version 4.1.1.; R Foundation for Statistical Computing, Vienna, Austria), and R package TwoSampleMR (https://github.com/MRCIEU/TwoSampleMR), and MR-PRESSO (https://github.com/rondolab/MR-PRESSO) (17).

## Results

The detailed characteristics of the SNPs associated with MS are displayed in **Supplementary Table 1**. The F-statistics of all 68 SNPs was above the threshold of 10, which indicated that they strongly predicted MS in the MR analysis.

**Figure 2:**
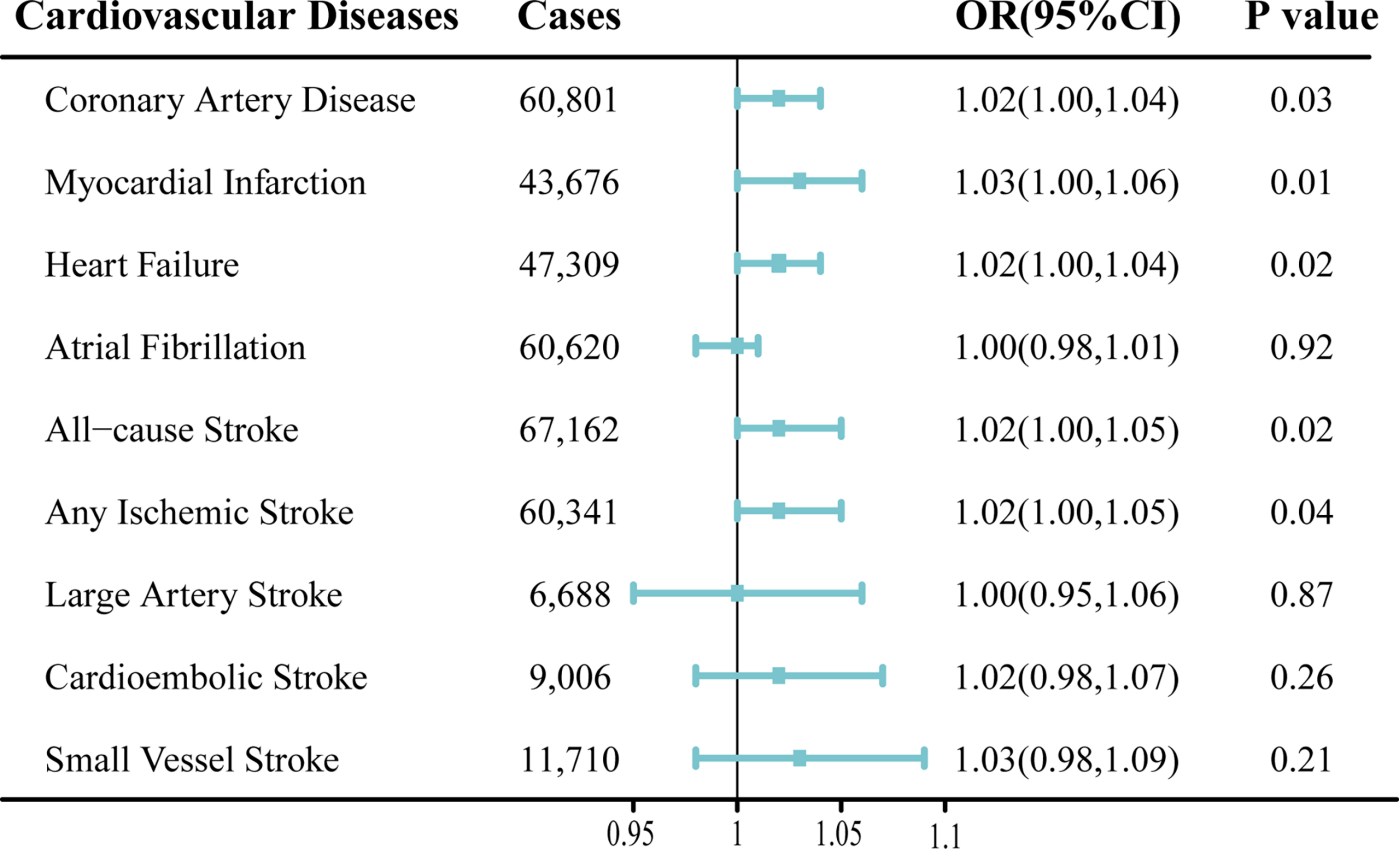
Mendelian randomization analysis estimates of multiple sclerosis and the risk of cardiovascular diseases. Odds ratios are scaled to per genetically predicted 1 log-odds unit increase in the liability to multiple sclerosis. OR, odds ratio; CI, confidence interval.

The results of the MR analysis estimates for the effect of MS on risk of CVDs are shown in **Figure 2**. In the IVW analyses, one unit increase in log odds of MS was suggestively associated with higher risks of CAD (OR, 1. 02; 95% CI, 1.00–1.04; *p* = 0.03), MI (OR, 1.03; 95% CI, 1.00–1.06; *p* = 0.01), HF (OR, 1.02; 95% CI, 1.00–1.04; *p* = 0.02), AS (OR, 1.02; 95% CI, 1.00–1.05; *p* = 0.02), and AIS (OR, 1.02; 95% CI, 1.00–1.05; *p* = 0.04) (**Figure 2**). However, no evidence was found for the causal associations between MS and the risk of AF (OR, 1.00; 95% CI, 0.98–1.01; *p* = 0.92) or other ischemic stroke subtypes (LAS: OR, 1.00; 95% CI, 0.95–1.06; *p* = 0.87; CES: OR, 1.02; 95% CI, 0.98–1.07; *p* = 0.26; SVS: OR, 1.03; 95% CI, 0.98–1.09; *p* = 0.21) (**Figure 2**). In addition, scatter plots visually displayed the associations between MS and subtypes of CVDs (**Supplementary Figures 1–9**).

The Weighted median method, MR-Egger regression, and MR-PRESSO method were performed as sensitivity analyses to reanalyze the suggestive associations observed in primary IVW analyses (**Table 2**). In the sensitivity analyses, the association patterns remained directionally consistent in most statistical models, and stable association was found in the MR-PRESSO analysis of MI (OR, 1.03; 95% CI, 1.01–1.05; *p* =0.02) (**Table 2**). Weak evidence of directional pleiotropy was found in the MR-Egger intercept tests (*p >*0.05; **Table 3**). However, the Cochrane’s Q test suggested that the main MR analyses suffered from different degrees of heterogeneity (**Table 3**). Nonetheless, we had more than 80% statistical power to detect the ORs for associations of MS with CAD (89%), MI (96%), HF (88%), AS (98%), and AIS (96%) (**Supplementary Table 2**).

**Table 2:** Sensitivity analyses of multiple sclerosis with cardiovascular diseases.

Outcome	Method	OR	95% CI	*p*-value
Coronary artery disease	Weighted median	1.02	0.95–1.09	0.24
	MR-Egger	0.95	0.85–1.06	0.38
	MR-PRESSO	1.02	1.00–1.05	0.05
Myocardial infarction	Weighted median	1.02	0.94–1.10	0.37
	MR-Egger	0.94	0.84–1.07	0.34
	MR-PRESSO	1.03	1.01–1.05	0.02
Heart failure	Weighted median	1.00	0.92–1.04	0.81
	MR-Egger	0.94	0.84–1.04	0.24
	MR-PRESSO	1.02	1.00–1.04	0.06
All-cause stroke	Weighted median	1.02	0.95–1.08	0.28
	MR-Egger	1.07	0.94–1.08	0.32
	MR-PRESSO	1.02	1.00–1.05	0.05
Any ischemic stroke	Weighted median	1.02	0.93–1.10	0.28
	MR-Egger	1.01	0.88–1.16	0.89
	MR-PRESSO	1.02	1.00–1.05	0.08

MR-PRESSO, Mendelian Randomization Pleiotropy Residual Sum and Outlier; OR, odds ratio; CI, confidence interval.

**Table 3:** Heterogeneity and pleiotropy tests for the associations of multiple sclerosis with cardiovascular diseases.

Outcome	Q-value	*P*_Q_	Intercept	*P*_intercept_
Coronary Artery Disease	77.81	0.132	0.010	0.167
Myocardial Infarction	49.91	0.517	0.011	0.158
Heart Failure	100.29	0.005	0.011	0.117
Atrial Fibrillation	100.60	0.002	0.000	0.996
All-cause Stroke	89.82	0.027	−0.005	0.525
Any Ischemic Stroke	91.72	0.020	0.002	0.844
Large Artery Stroke	89.32	0.030	0.018	0.398
Cardioembolic Stroke	82.46	0.083	−0.001	0.924
Small Vessel Stroke	89.18	0.030	−0.010	0.591

Q-value: the statistics of Cochrane’s Q test; P_Q_, p-value corresponding to Cochrane’s Q test; P_intercept_, p-value corresponding to MR-Egger intercept test.

## Discussion

In the current study, summary-level data from the large consortia and genetic studies were leveraged to investigate the causality of MS with CVDs. We found suggestive genetic evidence for the causal associations of genetic liability to MS with elevated risk of CAD, MI, HF, AS, and AIS, but not with AF or other stroke subtypes (LAS, CES, and SVS).

Results reported by several previous observational epidemiological studies on the associations between MS and CVDs were inconsistent and mixed. A meta-analysis, namely, 12 studies of 471,874 individuals indicated that compared with the general population, the risk of CVDs in patients with MS increased 29% (OR, 1.29; 95% CI, 1.80–2.51) (30). Another recent meta-analysis comprising 19 studies (44 to 66,616 participants) reported that the pooled OR estimate for MI was 1.41 in MS patients compared to the healthy controls (31). Besides, a meta-analysis of 9 observational studies suggested that MS was associated with an increased risk of MI and HF, but not ischemic heart disease or bradycardia (32). A population-based retrospective cohort study involving 12,251 MS patients and 72,572 controls showed that MS was associated with both acute coronary syndrome (HR, 1.28; 95% CI, 1.09–1.51) and cerebrovascular disease (HR, 1.59; 95% CI, 1.32–1.92) risk (7). Similarly, several retrospective cohort studies indicated MS led to a higher risk among MI, stroke, and HF (5, 6), which was in line with our MR results. Meanwhile, several prior studies also suggested that MS was associated with an increased risk of stroke (8, 33, 34). However, some other studies drew the inconsistent conclusion that MS was not a risk factor for some subtypes of CVDs. In a prospective cohort study, after adjusting for year and sex, the incidence of heart disease between MS patients and controls was no longer different [incidence rate ratio (IRR) 1.00; 95% CI, 0.94–1.06] (35). In addition, a population case–cohort study found no association between MS and ischemic heart disease and a 43% lower risk of AF compared with non-MS patients (10). Therefore, the current MR study was timely to draw attention to the assessment of the causality of MS with CVDs. A very recent two-sample MR study reported that MS was causally associated with an increased risk of MI (OR = 1.03), which was in line with our findings (36). However, this study found weak evidence for the causal association between MS and stroke using data from the CARDIoGRAMplusC4D consortium (40,585 stroke cases), while the genetic associations in the current study were obtained from the MEGASTROKE consortium involving 67,162 cases of all-cause stroke. On the one hand, the calculation of risk was according to the log odds of MS, which may partly explain the low values of ORs. On the other hand, there were much more healthy populations included in the outcome datasets in the current study, especially in the HF and the AF datasets.

The current two-sample MR study indicated suggestive causal associations between MS and a range of CVDs. Several underlying mechanisms have been proposed to explain the increased risk of CVDs in patients with MS. The causality found in the MR analysis was most likely due to common risk factors or etiological pathways, such as inflammation (37–39). Meanwhile, chronic pressure was associated with worsening disease in MS patients, which was related to a higher risk of stroke and MI (40, 41). Another speculative possibility was that immobilization and lack of physical activity amongst MS patients also increased the risk of CVDs (42). Besides, the immune-modulating treatments such as interferon β (INF-β) for MS patients were found to be associated with the risk of CVDs (43, 44). Other MS drugs like fingolimod were also reported to contribute to the risk of CVDs (45, 46). Previous studies have found that homocysteine plasma levels may be higher in patients with MS, which was a risk factor for CVDs (47–49). Other observational studies suggested endothelial dysfunction was evident in patients with MS (50, 51), which was a well-established risk factor of CAD (52).

The present study included several notable advantages. First, for the first time, MR analysis was employed to explore the causality of MS with a range of CVDs, which could largely reduce the influence of the environmental confounders and reverse causality. Second, the selected SNPs explained a relatively high proportion of MS. Furthermore, the large sample size of each MR analysis and the robust estimated effects of each instrumental variable (all F-statistics >10) ensure the statistical power in our study. Finally, several sensitivity analyses, such as the Weighted median analysis, MR-Egger regression, MR-PRESSO, and leave-one-out analysis were conducted to confirm the consistency of causal relationships.

However, several limitations should be considered when interpreting our findings. First, the influence of potential pleiotropy still could not be completely ruled out, though weak evidence of horizontal pleiotropy was found in the MR-Egger intercept tests and MR-PRESSO analyses. Second, different degrees of heterogeneity were detected in the Cochrane’s Q tests, indicating our analyses may be affected by pleiotropy. Third, MS patients in the current study were all of European descent, which might limit the generalizability of our findings to other populations. Further studies are needed to verify our findings in the non-European descents. Finally, we reported the relatively modest effect size (low values of ORs), thus the results of the current MR study should be interpreted with caution.

## Conclusion

In conclusion, this MR study provided suggestive genetic evidence for the causal associations of MS with increased risks of CAD, MI, HF, AS, and AIS. These findings highlight the significance of active monitoring and prevention of cardiovascular risk to combat cardiovascular comorbidities in MS patients. Further studies are required to illuminate the effectiveness of timely treating MS on reducing the risk of CVDs and investigate the potential mechanisms of these causal links. Considering the modest effect size, the results should be interpreted with caution.

## Data Availability Statement

The datasets presented in this study can be found in online repositories. The names of the repository/repositories and accession number(s) can be found in the article/**Supplementary Material**.

## Author Contributions

FY and TH designed the study and analyzed the data. FY drafted the manuscript. TH, JY, KH and HC interpreted the data and provided feedback on manuscript drafts. All authors listed have made a substantial, direct, and intellectual contribution to the work and approved it for publication.

## Funding

This work was supported by grants from the Key Laboratory of Precision Medicine for Atherosclerotic Diseases of Zhejiang Province, China (Grant No. 2022E10026), the Major Project of Science and Technology Innovation 2025 in Ningbo, China (Grant No. 2021Z134), and the Key Research and Development Project of Zhejiang Province, China (Grant No. 2021C03096).

## Conflict of Interest

The authors declare that the research was conducted in the absence of any commercial or financial relationships that could be construed as a potential conflict of interest.

## Publisher’s Note

All claims expressed in this article are solely those of the authors and do not necessarily represent those of their affiliated organizations, or those of the publisher, the editors and the reviewers. Any product that may be evaluated in this article, or claim that may be made by its manufacturer, is not guaranteed or endorsed by the publisher.
