# Supplementary material for: Multiple Sclerosis and the Risk of Cardiovascular Diseases: A Mendelian Randomization Study
Source: Front Immunol. 2022 Mar 15;13:861885. doi: 10.3389/fimmu.2022.861885 (PMC8964627; doi:10.3389/fimmu.2022.861885)

Supplementary Materials

Supplemental Table 1. Genome-wide significant (p<5×10^{-8}) single nucleotide polymorphisms that were used as instrument variables for multiple sclerosis.

SNP	chr	position	EA	OA	EAF	β	se	p value	r^2	F-statistics
rs10801908	1	117090493	C	T	0.23	0.215	0.026	3.5E-16	1.6E-02	1912.3
rs6670198	1	2520527	C	T	0.22	-0.145	0.018	2.0E-16	7.3E-03	846.1
rs1323292	1	192541021	A	G	0.98	0.124	0.022	1.4E-08	7.3E-04	84.3
rs35486093	1	85729820	A	G	0.15	-0.179	0.028	1.6E-10	8.0E-03	939.4
rs11809700	1	93152635	C	T	0.44	-0.144	0.018	3.5E-15	1.0E-02	1204.9
rs59655222	1	200875897	T	C	0.85	0.123	0.019	3.8E-11	3.8E-03	445.9
rs483180	1	120267505	G	C	0.90	-0.108	0.018	1.8E-09	2.1E-03	248.3
rs12133753	1	92222089	C	T	0.83	0.128	0.022	8.5E-09	4.6E-03	535.7
rs2317231	1	157686337	G	T	0.38	0.101	0.017	1.9E-09	4.8E-03	556.8
rs35540610	2	231121829	C	T	0.26	0.135	0.019	2.9E-12	7.0E-03	820.0
rs12478539	2	43355324	G	C	0.80	0.123	0.019	4.4E-11	4.8E-03	563.4
rs1177228	2	61242410	G	A	0.92	0.107	0.019	8.6E-09	1.6E-03	186.1
rs12622670	2	68646536	T	C	0.38	0.107	0.017	1.0E-10	5.4E-03	627.0
rs57116599	2	112770799	A	G	0.82	-0.120	0.020	2.6E-09	4.3E-03	496.5
rs438613	3	28072086	C	T	0.11	0.138	0.017	9.4E-17	3.7E-03	432.9
rs9843355	3	119228508	G	A	0.81	0.134	0.021	4.7E-10	5.5E-03	644.8
rs1014486	3	159691112	C	T	0.30	0.105	0.016	1.4E-10	4.6E-03	536.1
rs13327021	3	27783015	C	T	0.28	-0.113	0.017	4.5E-11	5.2E-03	603.2
rs10936602	3	169536637	T	C	0.76	0.108	0.019	1.7E-08	4.3E-03	498.3
rs4325907	3	101749022	T	C	0.46	-0.099	0.017	3.7E-09	4.9E-03	569.6
rs9992763	4	109058718	T	G	0.84	-0.090	0.016	4.5E-08	2.2E-03	256.2
rs10063294	5	35877505	A	G	0.21	-0.099	0.016	1.1E-09	3.2E-03	377.2
rs11749040	5	40396425	G	A	0.23	-0.197	0.023	3.5E-17	1.4E-02	1597.8
rs2546890	5	158759900	A	G	0.35	0.117	0.016	1.0E-12	6.2E-03	727.1
rs62420820	6	137438057	A	G	0.10	0.137	0.019	2.5E-13	3.5E-03	406.9
rs1738074	6	159465977	T	C	0.32	-0.114	0.017	9.9E-12	5.7E-03	659.0
rs4896153	6	135833463	A	T	0.33	-0.138	0.019	1.6E-13	8.5E-03	987.3
rs72928038	6	90976768	G	A	0.02	-0.161	0.025	9.0E-11	8.2E-04	95.5
rs2327586	6	135495226	T	C	0.61	0.118	0.019	7.8E-10	6.6E-03	766.7
rs802730	6	128280104	T	C	0.90	0.114	0.018	3.2E-10	2.3E-03	262.1
rs354033	7	149289464	G	A	0.78	0.108	0.019	1.2E-08	4.0E-03	464.3
rs55858457	7	2443302	G	T	0.45	-0.113	0.020	1.2E-08	6.3E-03	736.5
rs28703878	8	79417222	A	G	0.57	-0.134	0.021	4.5E-10	8.8E-03	1025.7
rs6990534	8	128814091	A	G	0.70	-0.107	0.018	3.6E-09	4.8E-03	561.9
rs7855251	9	100868189	T	C	0.38	0.110	0.020	4.2E-08	5.7E-03	666.8
rs11256593	10	6117322	T	C	0.13	0.186	0.017	6.8E-27	8.1E-03	941.1
rs1250551	10	81059335	G	T	0.12	-0.116	0.017	2.7E-11	2.9E-03	338.0
rs1112718	10	94479107	A	G	0.55	0.106	0.017	2.5E-10	5.5E-03	642.3
rs4939490	11	60793651	G	C	0.47	0.137	0.017	4.2E-15	9.3E-03	1086.9
rs6589706	11	118747813	A	G	0.07	0.104	0.017	2.2E-09	1.4E-03	164.9

rs12365699	11	118743286	G	A	0.98	0.144	0.023	3.1E-10	9.8E-04	114.0
rs1800693	12	6440009	T	C	0.35	-0.127	0.017	1.0E-13	7.4E-03	859.2
rs701006	12	58106836	G	A	0.50	0.114	0.017	1.3E-11	6.5E-03	755.5
rs7975763	12	123604053	C	T	0.28	-0.121	0.021	7.8E-09	5.9E-03	682.6
rs9591325	13	50811220	T	C	0.96	0.212	0.034	4.2E-10	3.2E-03	369.4
rs12434551	14	69253364	A	T	0.66	0.104	0.016	1.8E-10	4.8E-03	563.4
rs12147246	14	103265844	G	A	0.84	-0.099	0.017	4.3E-09	2.6E-03	305.9
rs34695601	14	76014298	T	C	0.99	0.109	0.020	3.2E-08	2.9E-04	33.5
rs6496663	15	90887584	A	C	0.67	-0.101	0.018	2.8E-08	4.5E-03	519.9
rs3809627	16	30103160	A	C	0.65	-0.097	0.018	3.2E-08	4.3E-03	499.8
rs12925972	16	79111297	C	T	0.48	0.095	0.017	3.1E-08	4.5E-03	519.1
rs35703946	16	86021505	G	A	0.89	0.173	0.029	1.9E-09	5.8E-03	677.7
rs405343	16	1067832	G	T	0.38	-0.118	0.022	4.7E-08	6.6E-03	769.2
rs17724508	16	79350204	T	C	0.96	0.213	0.038	2.6E-08	3.5E-03	411.6
rs2150879	17	57859210	G	A	0.90	0.104	0.016	3.3E-10	2.0E-03	231.3
rs1026916	17	40529835	G	A	0.64	-0.130	0.017	1.0E-13	7.7E-03	899.2
rs11079784	17	45702280	C	T	0.32	0.107	0.016	4.3E-11	5.0E-03	584.6
rs7222450	17	43407670	A	G	0.43	0.098	0.017	1.8E-08	4.8E-03	553.1
rs9955954	18	56348044	G	A	0.74	-0.110	0.019	1.5E-08	4.7E-03	542.3
rs1077667	19	6668972	C	T	0.88	0.152	0.021	8.4E-13	4.9E-03	566.4
rs4808760	19	18301979	G	C	0.81	-0.135	0.019	4.8E-13	5.6E-03	657.1
rs28834106	19	10592144	T	C	0.29	0.129	0.020	3.8E-11	6.9E-03	801.2
rs1465697	19	49837246	C	T	0.31	-0.124	0.019	3.5E-11	6.6E-03	769.2
rs11083862	19	47638539	A	T	0.81	0.101	0.017	4.1E-09	3.1E-03	364.5
rs2248137	20	52789743	G	C	0.26	-0.112	0.017	7.8E-11	4.9E-03	565.3
rs6032662	20	44734310	C	T	0.02	0.134	0.018	2.8E-13	7.8E-04	90.8
rs140522	22	50971266	C	T	0.53	-0.111	0.018	2.8E-10	6.1E-03	709.9
rs9610458	22	22205353	T	C	0.35	0.114	0.017	4.6E-12	5.9E-03	693.0

Supplemental Table 2. The statistical power of Mendenlian randomization analyses of multiple sclerosis with cardiovascular diseases.

**Outcome**	**Coronary artery disease**	**Myocardial infarction**	**Heart failure**	**All-cause stroke**	**Any ischemic stroke**
**Sample size**	184,305	171,875	977,323	521,612	521,612
**Type-I error rate**	0.05	0.05	0.05	0.05	0.05
**Proportion of cases**	0.33	0.25	0.05	0.13	0.12
**Odds ratio of outcome**	1.023	1.030	1.021	1.024	1.023
r^2	0.48	0.48	0.48	0.48	0.48
**Statistical power**	89%	96%	88%	98%	96%

r^2, proportion of variance explained for the association between the SNPs and the exposure variable.

Supplemental Table 3. Detailed data of the main Mendelian randomization estimates.

Cardiovascular Diseases	Estimate	SE	OR	LCI	UCI	*p*
Coronary Artery Disease	0.023	0.010	1.023	1.002	1.044	0.029
Myocardial Infarction	0.029	0.012	1.030	1.007	1.053	0.011
Heart Failure	0.021	0.009	1.021	1.003	1.039	0.019
Atrial Fibrillation	-0.001	0.008	0.999	0.984	1.015	0.922
All-cause Stroke	0.024	0.010	1.024	1.004	1.045	0.020
Any Ischemic Stroke	0.023	0.011	1.023	1.001	1.046	0.038
Large Artery Stroke	0.004	0.028	1.004	0.952	1.060	0.872
Cardioembolic Stroke	0.024	0.021	1.024	0.982	1.068	0.258
Small Vessel Stroke	0.032	0.026	1.032	0.982	1.085	0.215

Estimate, the Mendelian randomization estimates for the associations between multiple sclerosis and cardiovascular diseases. SE, standard error; OR, odds ratio; LCI, lower confidence interval; UCI, upper confidence interval.

Figure Legend

Supplemental figure 1. The scatter plot for the association between multiple sclerosis and coronary artery disease.

Supplemental figure 2. The scatter plot for the association between multiple sclerosis and myocardial infarction.

Supplemental figure 3. The scatter plot for the association between multiple sclerosis and heart failure.

Supplemental figure 4. The scatter plot for the association between multiple sclerosis and atrial fibrillation.

Supplemental figure 5. The scatter plot for the association between multiple sclerosis and all-cause stroke.

Supplemental figure 6. The scatter plot for the association between multiple sclerosis and any ischemic stroke.

Supplemental figure 7. The scatter plot for the association between multiple sclerosis and large artery stroke.

Supplemental figure 8. The scatter plot for the association between multiple sclerosis and cardioembolic stroke.

Supplemental figure 9. The scatter plot for the association between multiple sclerosis and small vessel stroke.

Supplemental figure 1

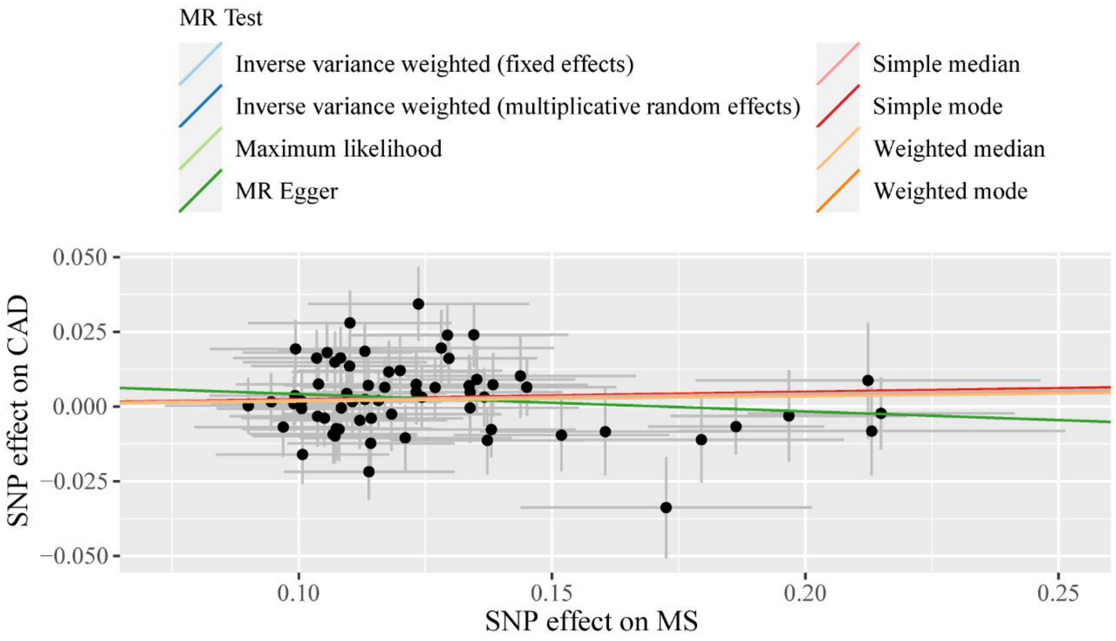

Supplemental figure 2

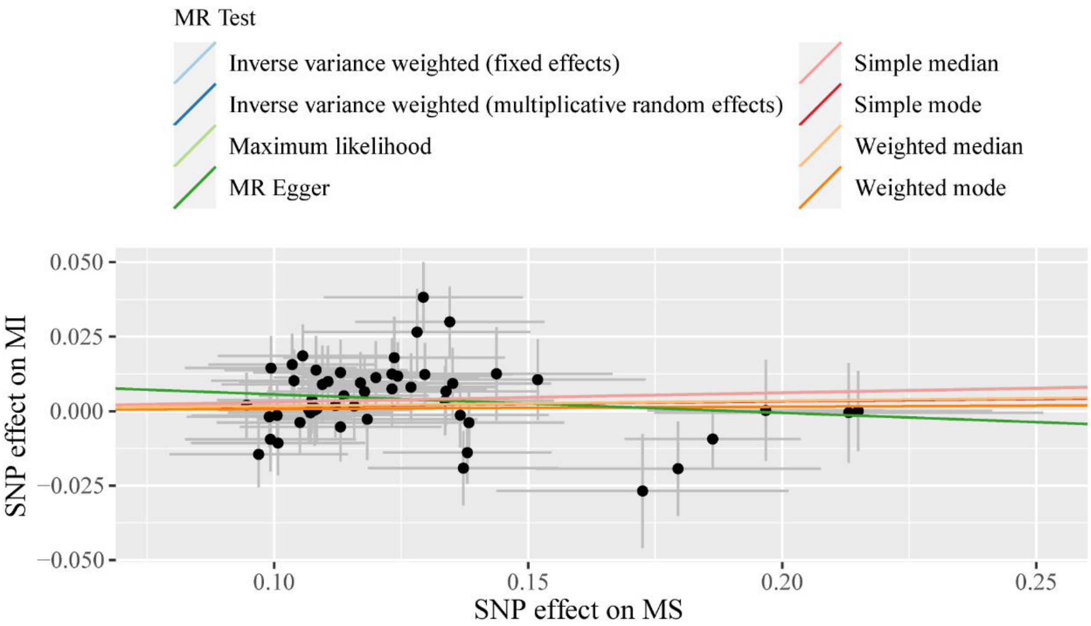

Supplemental figure 3

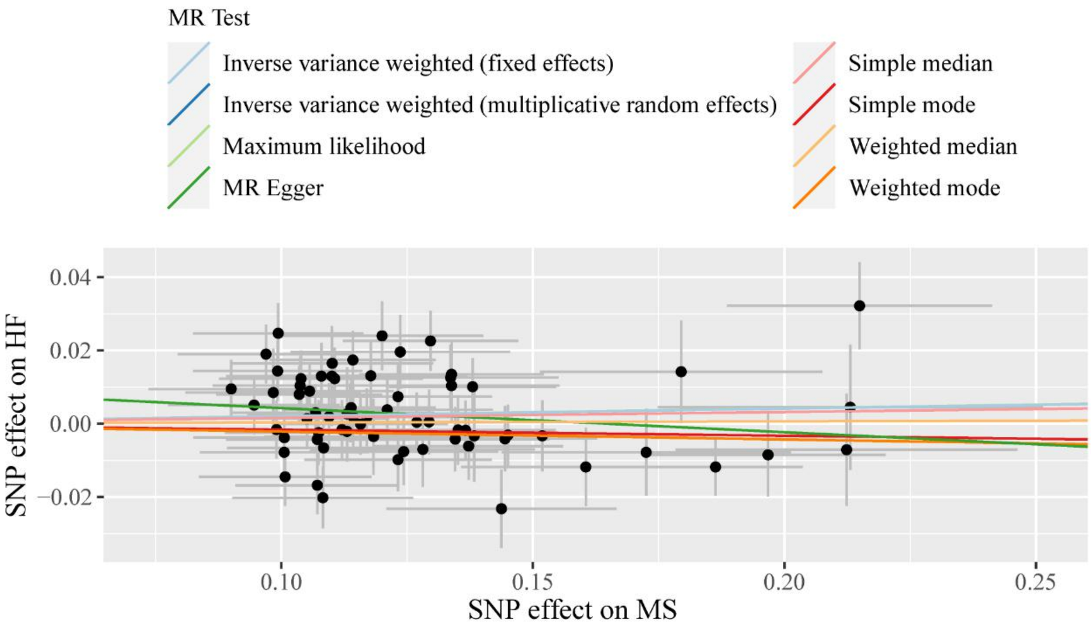

Supplemental figure 4

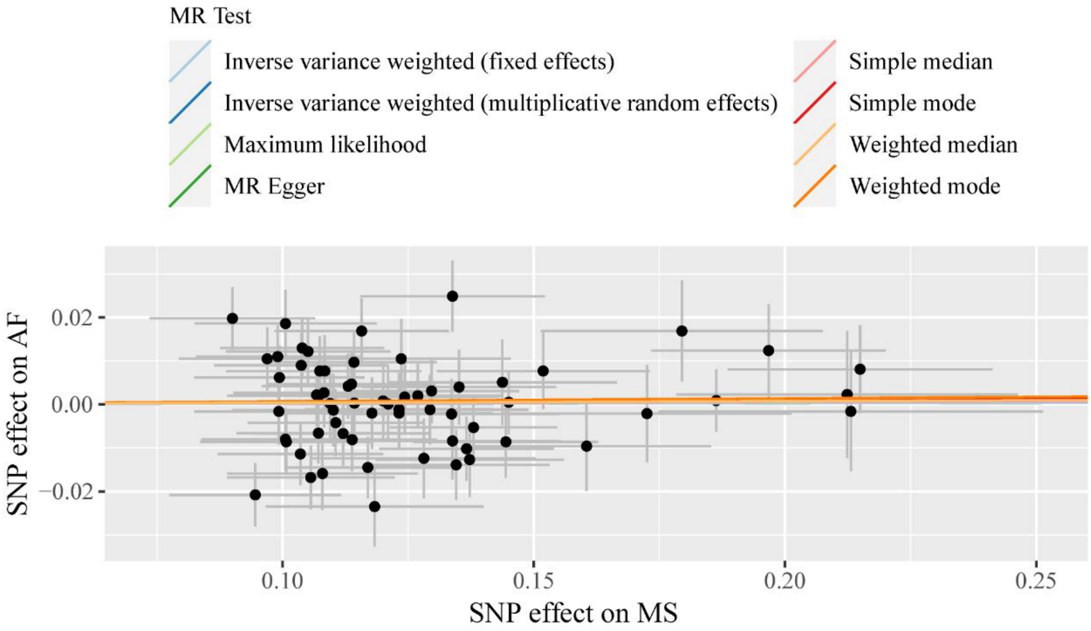

Supplemental figure 5

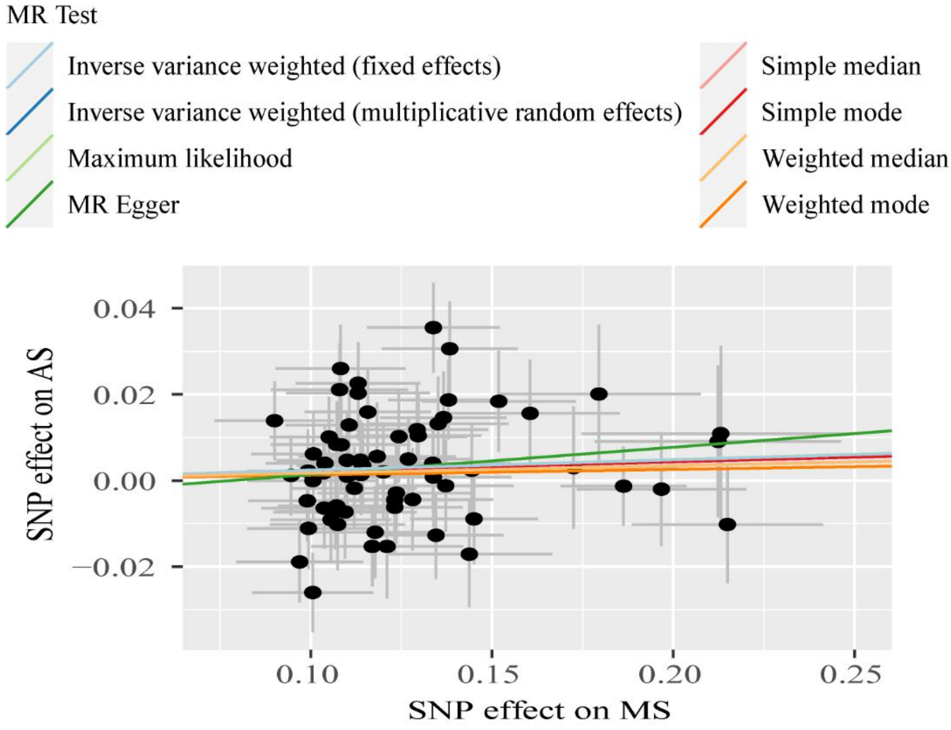

Supplemental figure 6

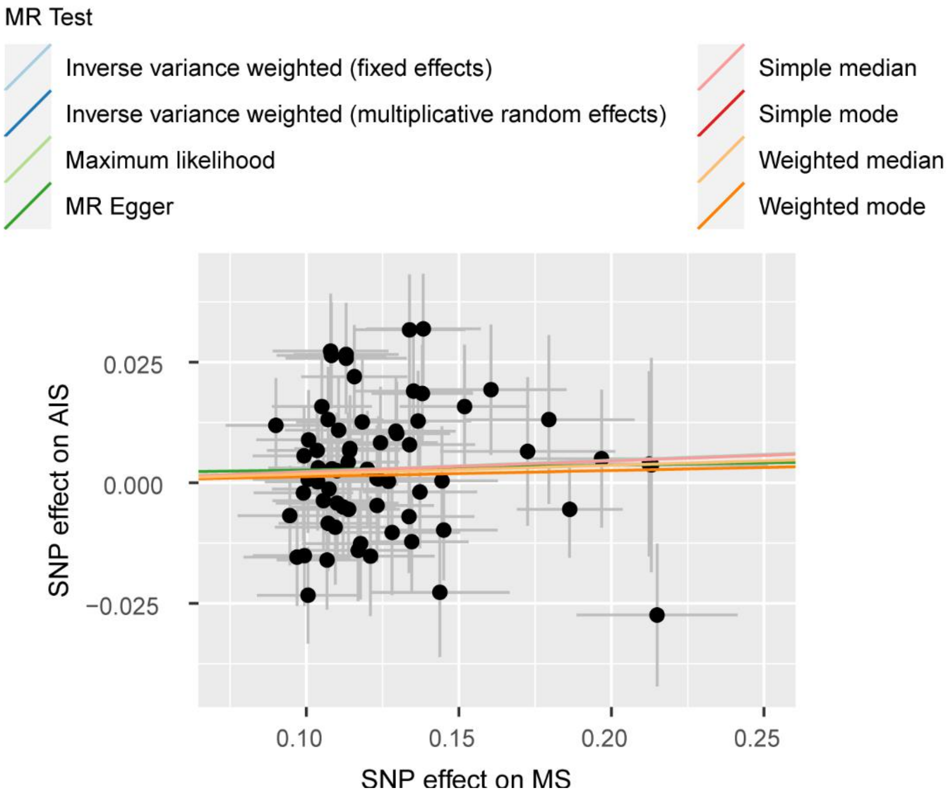

Supplemental figure 7

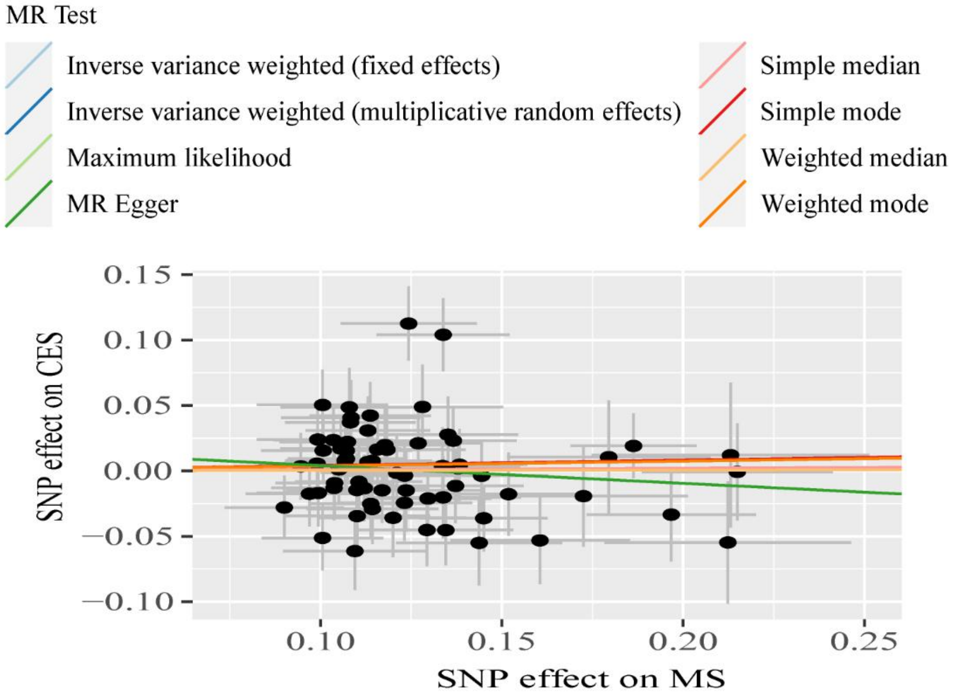

Supplemental figure 8

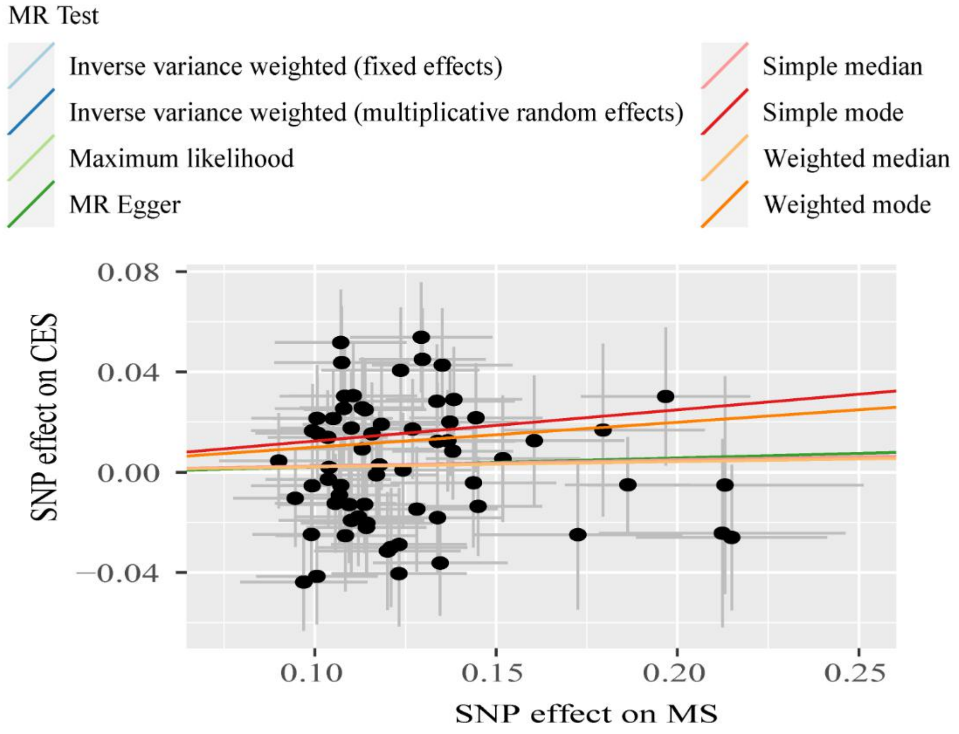

Supplemental figure 9

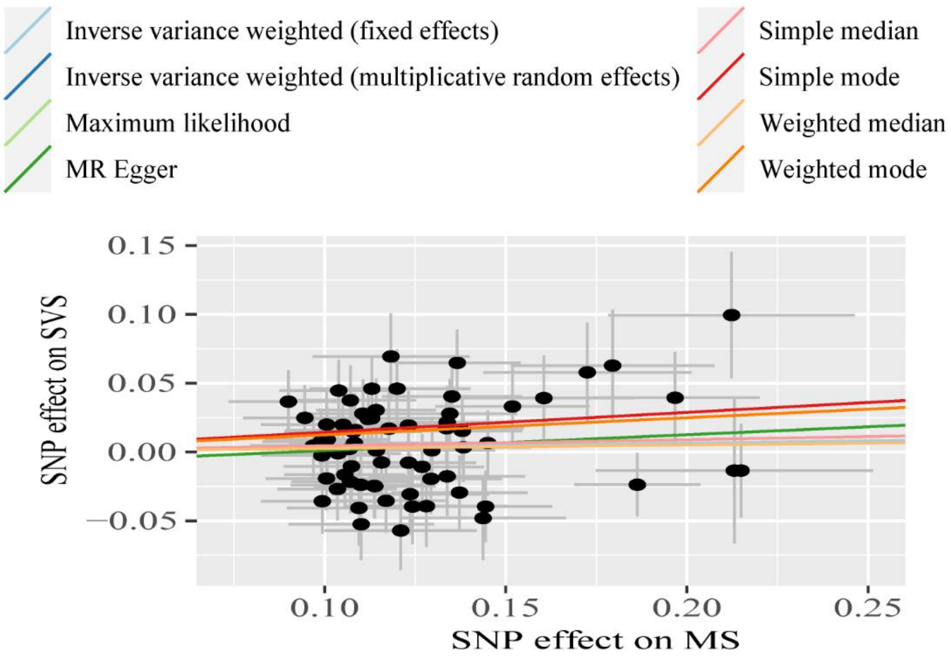

Supplement: Supplementary file 1 [file DataSheet_1.pdf]
